# Patterns of the Non-Indigenous Isopod *Cirolana harfordi* in Sydney Harbour

**DOI:** 10.1371/journal.pone.0086765

**Published:** 2014-01-22

**Authors:** Ana B. Bugnot, Ross A. Coleman, Will F. Figueira, Ezequiel M. Marzinelli

**Affiliations:** 1 School of Biological Sciences, The University of Sydney, Sydney, New South Wales, Australia; 2 Sydney Institute of Marine Science, Mosman, New South Wales, Australia; 3 School of Biological, Earth and Environmental Sciences, University of New South Wales, Sydney, New South Wales, Australia; University of Western Sydney, Australia

## Abstract

Biological introductions can alter the ecology of local assemblages and are an important driver of global environmental change. The first step towards understanding the impact of a non-indigenous species is to study its distribution and associations in the invaded area. In Sydney Harbour, the non-indigenous isopod *Cirolana harfordi* has been reported in densities up to 0.5 individuals per cm^2^ in mussel-beds. Abundances of this species have, however, been largely overlooked in other key habitats. The first aim of this study was to evaluate the abundances and distribution of *C. harfordi* across different habitats representative of Sydney Harbour. Results showed that *C. harfordi* occurred in oyster and mussel-beds, being particularly abundant in oyster-beds. We also aimed to determine the role of *C. harfordi* as a predator, scavenger and detritus feeder by investigating the relationships between densities of *C. harfordi* and (i) the structure of the resident assemblages, and (ii) deposited organic matter in oyster-beds. Densities of *C. harfordi* were not related to the structure of the assemblages, nor amounts of deposited organic matter. These findings suggested little or no ecological impacts of *C. harfordi* in oyster-beds. These relationships may, however, affect other variables such as growth of individuals, or be disguised by high variability of assemblages among different locations. Future studies should, therefore, test the impacts of *C. harfordi* on the size of organisms in the assemblage and use manipulative experiments to control for spatial variation. This study is the first published work on the ecology of the invasion of *C. harfordi* and provides the starting-point for the study of the impacts of this species in Sydney Harbour.

## Introduction

Biological introductions have caused environmental changes in many habitats by altering the composition and ecology of local assemblages [1]–[5], contributing to extinctions which ultimately may lead to homogenisation of biodiversity at a global scale [6]–[8], and increase in diversity by facilitating resident organisms at a local scale [8]–[10]. For example, the introduction of the kelp *Undaria pinnatifida* on the coast of Argentina provided a novel complex habitat, increasing richness and abundances of local species [11]. In spite of their potential for ecological impacts, the effects of many non-indigenous species (NIS) are still unknown [12], [13]. This study is focused on one of these unexplored species, the non-indigenous isopod *Cirolana harfordi* (Lockington, 1877) in Sydney Harbour, Australia.

**Figure 1:**
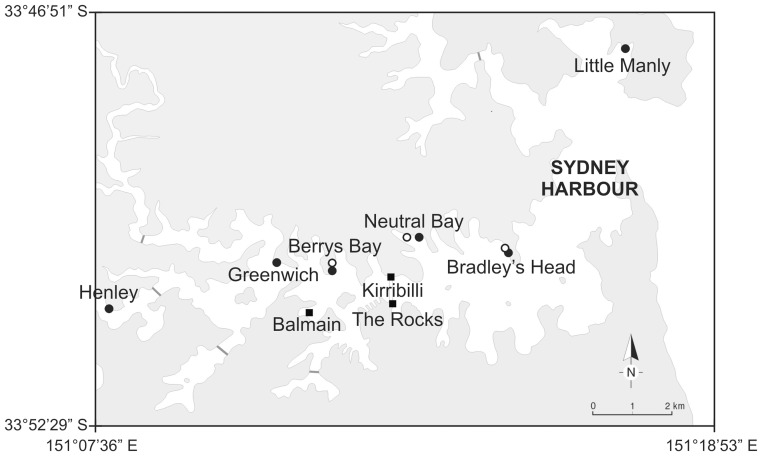
Locations sampled. Locations sampled in Sydney Harbour for oyster-beds (black circles), mussel-beds (squares) and coralline turfs (white circles).

*C. harfordi* is native to the North Pacific and is one of the most common littoral isopod species in California [14]. It has been reported to be a detritus feeder [15], a scavenger and an active predator that feeds on small polychaetes and other crustaceans [14]. To date, this species has only been described as non-indigenous in Australia and New Zealand [16]. It was first found in Australia in Berrys Bay (Sydney Harbour, New South Wales, Figure 1) in 1972, and then in Fremantle, Western Australia, and Lorne, Victoria [17].

In order to evaluate the effects of NIS, we first need to understand their distribution and associations. There is, however, little information about the patterns of habitat distribution and abundances of *C. harfordi* in Australia, and the only work done is an unpublished PhD dissertation [18]. This study found that *C. harfordi* had a broad distribution in Sydney Harbour, from Balmain to Little Manly, with peak abundances in the area of Berrys Bay (Figure 1). It was also found that *C. harfordi* occurred in mussel-beds and oyster-beds in Sydney Harbour, reaching densities of up to 0.5 individuals per cm^2^ in mussel-beds [18]. Other representative habitats in the area were not investigated. In contrast, in its native range, *C. harfordi* was reported to occur in large abundances in a variety of biogenic habitats, such as macroalgae (*Pelvetia* sp. and *Fucus* sp.), inside the tubes of polychaetes, in mussel-beds and amongst dead shells of barnacles [14], [15]. It was found to reach densities of over 20 individuals per cm^2^ among tubeworms and 1.4 individuals per cm^2^ in mussel-beds. Therefore, the first aim of this study was to determine the abundance and distribution of *C. harfordi* across different biogenic habitats in Sydney Harbour.

Highly abundant NIS, such as *C. harfordi*, are expected to have strong effects on resident assemblages via interactions with other organisms. Based on population theory [19], [20], it is expected that organisms which strongly interact with each other will have abundances that are correlated. As a predator, *C. harfordi* may affect resident assemblages by establishing strong, negative biological interactions with their prey. In addition, generalist predators in enclosed areas, as in the case of *C. harfordi* in Sydney Harbour, are expected to affect the survival and/or growth of organisms in resident assemblages [21]. Thus, the second aim of this study was to determine the relationships between the densities of *C. harfordi* and the abundance and number of taxa of the resident assemblages.

Additionally, as a scavenger and a detritus feeder, *C. harfordi* can indirectly influence the habitat by changing deposited organic matter and carbon cycling, thereby affecting availability of resources for the organisms in the assemblage [22]. Therefore, the third aim of this study was to determine the relationship between the densities of *C. harfordi* and the amount of deposited organic matter in oyster-beds. As organic matter and densities of *C. harfordi* could also be related to the thickness of the oyster-beds (A. Bugnot, personal observation), thickness of the oyster-bed was also quantified.

This study explored the habitat distribution, abundances and associations of *C. harfordi* in Sydney Harbour by doing a series of observational experiments. We tested the hypotheses that the densities of *C. harfordi* in Sydney Harbour will (i) differ between biogenic habitats, (ii) be related to the structure of the resident assemblages and (iii) correlate with the quantity of deposited organic matter and thickness of the habitat. This is the first published study of the ecology of the invasion of this species and thus provides the baseline for future studies on the topic.

## Methods

### Ethics statement

The work described in this paper conforms to the legal requirements of Australia, including those relating to conservation and welfare, and to the University of Sydney's Policies on Ethical and Responsible Behaviour in Research. Procedures and protocols for the use of animals in research at the University of Sydney are mandated by state legislation from the New South Wales Parliament. Studies involving invertebrates, except on Cephalopoda, are exempt from requirements to submit protocols for ethics approval. Collection of animals was done under New South Wales Fisheries research permit F96/146-7.1–2.

### Abundance *of C. harfordi* in different habitats

Mussel-beds (*Mytilus* sp.), oyster-beds (a mix of the native Sydney Rock oyster *Saccostrea glomerata*, and the non-indigenous Pacific oyster *Crassostrea gigas*) and patches of coralline turfs (*Corallina officinalis*), which are common intertidal habitats in Sydney Harbour [23], [24], were sampled in June 2010. The locations sampled in this first experiment were situated in the area where *C. harfordi* was reported to be most abundant [18]. A hierarchical sampling design was used: for each habitat, three locations (separated by 1–2 km, Figure 1) were randomly chosen (except for mussel-beds; see below) and, within each location, three patches (separated by 1–2 m) were randomly selected. Three haphazardly selected replicates were taken in each patch (separated by 10–20 cm). Oyster-beds and coralline turfs were sampled on the rocky shores at Berrys Bay, Neutral Bay and Bradley's Head (Figure 1). Mussel-beds were sampled on seawalls at Balmain, The Rocks and Kirribilli (Figure 1), the only three locations where this habitat was found at the time (A. Bugnot, personal observation).

To sample oyster-beds, an area of approximately 10 cm in diameter was sampled using a chisel and a hammer. When sampling mussel-beds and coralline turfs, samples were collected using a 10-cm diameter corer and a scraper. Samples were transported to the laboratory and preserved in 7% formalin. The volume of each sample was estimated by submersing the samples in 1 L of water and calculating the displaced volume. This was done for all habitats. Individuals of *C. harfordi* were identified and counted. *C. harfordi* were identified based on the description by Bruce [17], and distinguished from *Eurylana arcuata*, a cirolanid present in the same area, based on the differences in the morphology of the clypeus, as described by Bowman et al. [25]. Even though the area sampled is approximately the same, different habitats have different structural complexity and different locations of the same habitat may present different thickness. Therefore, to compare the results of this study among habitats and locations, densities were estimated as number of individuals per 100 ml of sample. However, to compare abundances with other studies, numbers of individuals per cm^2^ were used instead, which were calculated by considering that cores were circular and with a diameter of 10 cm. The densities of *C. harfordi*, transformed as ln (x+1), were compared among habitats with ANOVA using GMAV 5 (EICC, The University of Sydney) with the assumption of homogeneity of variances examined using Cochran's test [26]. Interactions with a *p*-value greater than 0.25 were eliminated to increase the power for factors higher in the table [26].

### Relationships between *C. harfordi* and the resident assemblage in oyster-beds

Based on the large abundances of *C. harfordi* recorded in oyster-beds (see Results section), the rest of the study focussed on this habitat. To examine the relationships between the densities of *C. harfordi* and the structure of the resident assemblage, samples of oyster-beds collected in June 2010 were rinsed using a 500 µm sieve and the organisms retained were identified to the finest taxonomic resolution possible, either Species, Genus, Family (for polychaetes) or morpho-species (for amphipods). Previous studies in Sydney Harbour have found no differences in the patterns of resident assemblages whether identification is at the species or morpho-species level [27]. In addition, groups such as nemerteans, nematodes and oligochaetes (which were found in low abundances) were not identified further than Order or Phyla because of lack of taxonomic information.

The structure of the assemblage was evaluated using multivariate (densities of individuals per taxa) and univariate metrics (total abundance and number of taxa, each standardised per 100 ml of sample). The number of taxa was not significantly correlated with total abundances at any location (Pearson Product Moment Correlation for Berrys Bay *r* = 0.43, Neutral Bay *r* = −0.43 and Bradley's Head *r* = 0.51; df = 8; *p*>0.05 at all three locations), hence no individual-based standardisation was necessary [28]. The relationships between the densities of *C. harfordi* and the structure of the assemblage were examined doing PERMANOVA (9999 permutations of residuals under reduced model) on the densities per taxa, total abundance and number of taxa using the density of *C. harfordi* as a covariate [29]. The patterns of these relationships over all locations were tested by the effect of the covariate densities of *C. harfordi*, whereas differences between locations were tested by the interaction between densities of *C. harfordi* and location. The multivariate metric was forth-root transformed and analyses were done on Bray-Curtis similarities. The univariate metrics were analysed using Euclidean distances. Interactions with a *p*-value greater than 0.25 were eliminated to increase the power for factors higher in the table [26].

This study was repeated in October 2010, but this time oyster-beds on rocky shores were sampled at six locations, extending outside the known range of distribution of *C. harfordi* in Sydney Harbour. In this second survey, the same three locations (Berrys Bay, Neutral Bay and Bradley's Head) were sampled as above with the addition of Henley, Greenwich and Little Manly (Figure 1). At each location, nine samples were haphazardly taken. As the previous sampling showed no significant differences in the densities of *C. harfordi* among patches (ANOVA, *F*_18,54_ = 1.08, *p*>0.05), this factor was not included in the design. Samples were collected and processed and data analysed as described above.

### *C. harfordi,* organic matter and thickness of the habitat

Ten samples of oyster-beds were collected at Berrys Bay and Greenwich (Figure 1), as described above. The thickness of the bed was estimated by hammering a chisel into the oyster-bed until it reached the rock, and measuring the depth to the substratum. The samples were taken to the laboratory and preserved at −20°C for two days. Samples were then submersed in 1 L of deionised water and the displaced volume measured. Oysters were brushed in the same water to collect all the deposited material. This solution was then filtered using 500 µm and 64 µm sieves. At this stage, known volumes of deionised water were added to help the sieving process. *C. harfordi* individuals, retained in the 500 µm sieve, were counted. Organic matter (OM) was quantified for the fraction smaller than 64 µm and the fraction between 64 and 500 µm. These fractions were separated because the fraction between 64–500 µm contains organic matter not only in the form of particles, but also as meiofauna. For the 64–500 µm fraction, the whole sample was processed. For the fraction smaller than 64 µm, the collected volume of wash was high (between 2 and 4 L), so three subsamples of 100 ml were processed for analyses. To quantify OM on these samples, the loss on ignition technique was used as described by Luczak et al. [30], by drying the samples at 60°C for 48 h and then igniting them at 500°C for 5 h. Numbers of individuals of *C. harfordi* and weight of OM were standardised per 100 ml of sample.

Pearson correlations were done to compare the thickness of the habitat with the concentration of OM for the fraction smaller than 64 µm and concentration of OM for the fraction between 64–500 µm. To examine the relationships between the densities of *C. harfordi* and the predictor variables location, thickness of the habitat, concentration of OM for the fraction smaller than 64 µm and concentration of OM for the fraction between 64–500 µm, a DISTLM analysis was done in PERMANOVA 6 [31]. Predictor variables were first analysed individually for their relationships with the densities of *C. harfordi* and were then subjected to a step-forward selection procedure, starting with the predictor variable that explained the highest percentage of the variation and finishing with the one with the lowest [32]. Thickness and concentration of OM were normalized and Euclidean distances were used for the analyses.

## Results

### Abundance of *C. harfordi* in different habitats

**Figure 2:**
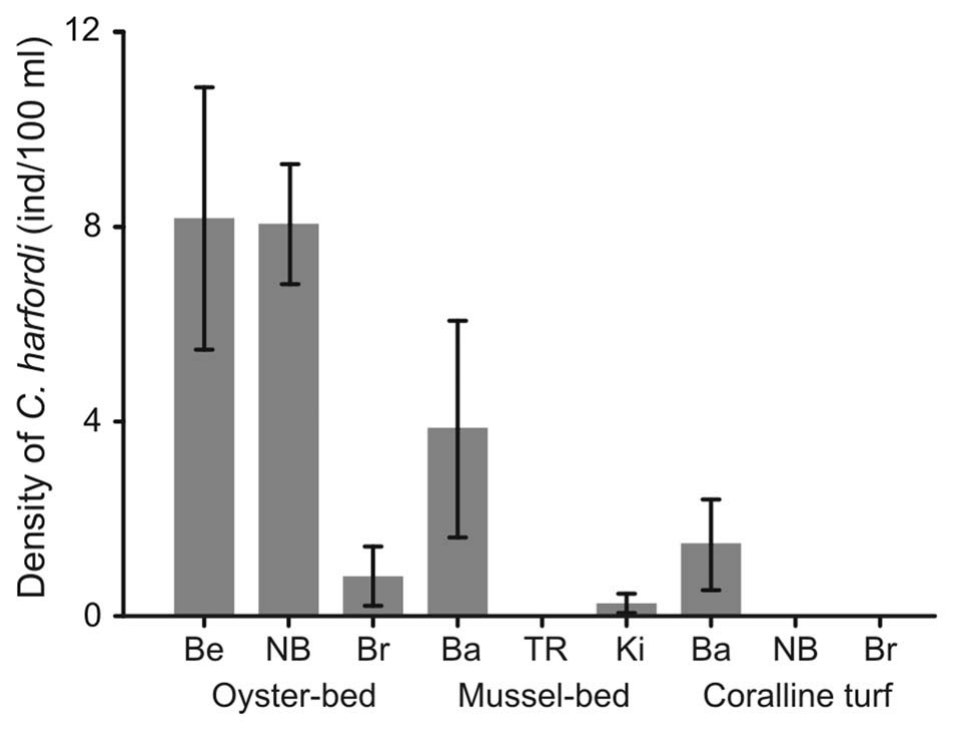
Habitat distribution and densities. Mean (± S.E.) densities of *Cirolana harfordi* for oyster-beds, mussel-beds and coralline turfs at Berrys Bay (Be), Neutral Bay (NB), Bradley's Head (Br), Balmain (Ba), The Rocks (TR) and Kirribilli (Ki) in June 2010.

The densities of *C. harfordi* did not differ significantly among oyster-beds, mussel-beds and coralline turfs in June 2010 (Figure 2, ANOVA, *F*_2,6_ = 3.14, *p*>0.05). Densities of *C. harfordi* in oyster-beds were, however, considerable greater than in mussel-beds and coralline turfs. Mean densities in oyster-beds, between the three locations sampled, were 5.7 ± SE 2.4 individuals/100 ml of sample, compared to 1.4 ± SE 1.2 in mussel-beds and 0.5 ± SE 0.5 in coralline turfs (Figure 2).

### Densities of *C. harfordi* in oyster-beds and its relative abundance in the assemblage

**Figure 3:**
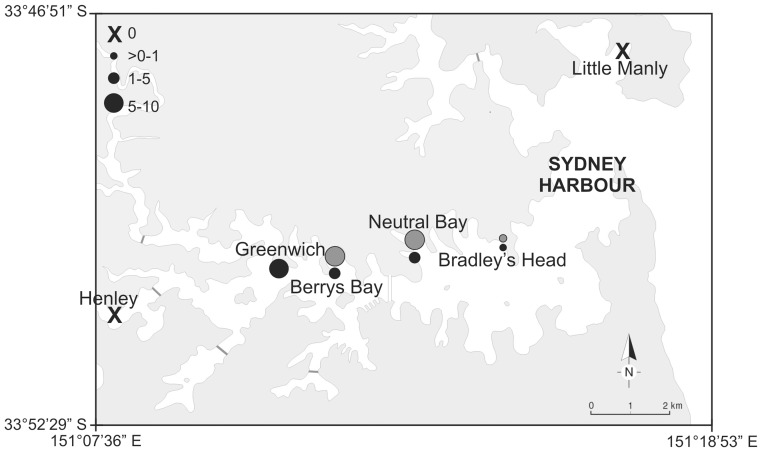
Distribution along Sydney Harbour. Mean densities of *Cirolana harfordi* (ind/100 ml of sample) in oyster-beds in Sydney Harbour. Berrys Bay, Neutral Bay and Bradley's Head were sampled in June (grey) and October (black) 2010. Henley, Greenwich and Little Manly were sampled only in October 2010 (see methods). There were no *C. harfordi* individuals found at Henley and Little Manly.

There were significant differences in the densities of *C. harfordi* in oyster-beds between locations in June and October 2010 (Figure 3, ANOVA, June *F*_6,54_ = 3.9, *p*<0.05; October *F*_5,48_ = 12.65, *p*<0.05). *C. harfordi* was abundant at Greenwich (October 8.4 ± SE 1.0 individuals/100 ml), Berrys Bay (June 8.2±1.0, October 2.2± SE 0.3 individuals/100 ml) and Neutral Bay (June 8.1±0.5, October 4.5 ± SE 0.6 individuals/100 ml) and was absent at Henley and Little Manly (Figure 3). Bradley's Head had few *C. harfordi* (June 0.8±0.2, October 0.5 ± SE 0.1 individuals/100 ml). At locations where it was most abundant in June and October 2010, *C. harfordi* was between the third and tenth group in density among over 70 taxa and it was one of the most abundant arthropods, together with a Chironomidae larvae and the isopod *Dynoides barnardii* (Table 1).

**Table 1:** Densities of *Cirolana harfordi* (mean ± S.E. number of individuals in 100 ml of oyster-bed) compared with densities of the most abundant arthropods (Chironomidae larvae and *Dynoides barnardii*) at Greenwich, Berrys Bay and Neutral Bay in June and October 2010. Greenwich was not sampled in June 2010 (see methods).

Location	Greenwich		Berrys Bay		Neutral Bay
**June 2010**					
*C. harfordi*			8.17±0.85	8.05±0.39	
Chironomidae larvae			4.69±0.51	1.07±0.23	
*D. barnardii*			31.86±5.39	2.24±0.18	
**October 2010**					
*C. harfordi*	8.36±2.74		2.21±0.83	4.52±1.70	
Chironomidae larvae	12.84±2.49		3.42±0.32	4.28±0.98	
*D. barnardii*	47.23±13.23		1.74±0.55	3.88±1.94	

### Relationships between *C. harfordi* and the resident assemblage in oyster-beds

There was no significant relationship between the densities of *C. harfordi* and the densities per taxa, total abundance and number of taxa in the resident assemblage in oyster-beds (Table 2). These results were consistent between the samples taken in June and October 2010. There was, however, a trend for a positive relationship between the densities of *C. harfordi* and the number of taxa in October (Table 2).

**Table 2:** Analyses of the effects of the densities of *Cirolana harfordi* on densities per taxa, total abundance and number of taxa of the assemblage in oyster-beds in June and October 2010. PERMANOVA (9999 permutations of residuals under reduced model) of Bray-Curtis similarities for the densities per taxa (forth root transformed) and Euclidean distances for total abundance and number of taxa in oyster-beds, where Location is the comparison among three locations in June and six locations in October 2010 (random) and Patch is the comparison among three patches per location (random, nested in Location), using the densities of *C. harfordi* as a covariate. Factor Patch was not included in the analysis in October 2010 (see methods). ^a^ Terms eliminated to increase the power for factors higher in the table.

		Densities per taxa			Total abundance			Number of taxa		
Source	df	MS	Pseudo-*F*	*p* (perm)	MS	Pseudo-*F*	*p* (perm)	MS	Pseudo-*F*	*p* (perm)
June 2010										
Location Lo	2	3829	2.54	0.0001	56222	0.61	0.59	266.1	4.23	0.05
Patch Pa (Lo)	6	1506	2.16	0.0002	92556	5.06	0.006	62.8	2.37	0.08
*C. harfordi* Ci	1	527	0.76	0.67	37810	2.07	0.16	77.9	2.93	0.11
Ci x Lo	2	757	^a^		6135	^a^		9.9	^a^	
Ci x Pa (Lo)	5	777.	^a^		21022	^a^		31.4	^a^	
Residual	10	643			19368			27.4		
October 2010										
Location Lo	5	5702	9.37	0.0001	1390000	15.71	0.0001	107.3	2.94	0.02
*C. harfordi* Ci	1	962	1.58	0.12	1722	0.02	0.86	141.5	3.88	0.051
Ci x Lo	3	342	^a^		17293	^a^		16.6	^a^	
Residual	44	626			93647			37.8		

### *C. harfordi,* organic matter and thickness of the habitat

**Figure 4:**
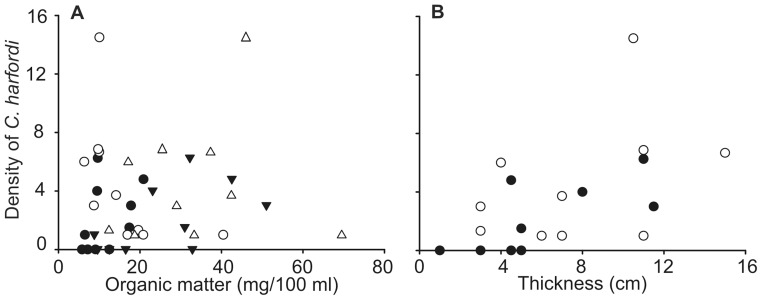
Relationships between *C. harfordi* and organic matter and thickness of the habitat. Densities of *Cirolana harfordi* and (A) concentration of organic matter smaller than 64 µm (triangles) and 64–500 µm (circles) and (B) thickness of the oyster-bed at Greenwich (white) and Berrys Bay (black)

Thickness of the oyster-bed was positively corrected with the concentration of OM in the fraction smaller than 64 µm (Pearson correlation, *r* = 0.60, df = 19, *p*<0.05), but not correlated with the fraction between 64 and 500 µm (Person correlation, df = 19, *p*>0.05). Thickness of the oyster-bed was significantly correlated with the densities of *C. harfordi*, but concentration of OM in the fraction smaller than 64 µm and the fraction between 64 and 500 µm were not related to the densities of *C. harfordi* (Table 3, Figure 4).

**Table 3:** DISTLM of the densities of *Cirolana harfordi* in oyster-beds, using Thickness of the habitat, Concentration of organic matter smaller than 64 µm (OM<64), Concentration of organic matter between 64 and 500 µm (OM 64–500) and Location as predictor variables. For marginal tests, variables are taken individually; for sequential tests, variables were chosen using Forward selection criteria. The column %var indicates the percentage of the variation explained by the predictor variables. Cum % is the cumulative percentage of variance explained.

Variable	Pseudo-*F*	*p*	% var	Cum %
**Marginal tests**				
Thickness	7.15	0.013	28.4	
Location	2.57	0.12	12.5	
OM<64	2.09	0.16	10.4	
OM 64–500	0.79	0.35	4.2	
**Sequential tests**				
Thickness	7.15	0.015	28.4	28.4
Location	1.41	0.26	5.5	33.9
OM 64–500	2.44	0.13	8.7	42.6
OM<64	0.09	0.76	0.3	43.0

## Discussion

Although the differences between habitats were not significant, *C. harfordi* occurred in great densities in oyster-beds and mussel-beds, and only a few individuals were found in coralline turfs in Sydney Harbour. The abundances reached in oyster-beds were an order of magnitude greater than in other habitats. In oyster-beds, *C. harfordi* had a mean density of 0.1 ± SE 0.03 individuals/cm^2^ and reached densities of up to 0.54 individuals/cm^2^, compared to an average of 0.03 ± SE 0.02 and up to 0.33 individuals/cm^2^ in mussel-beds (data for mussel-beds calculated using the two locations where *C. harfordi* was found). Although *C. harfordi* was one of the most abundant taxa in oyster-beds, these densities were not as large as those reported in the literature for Japan and California, where up to 1.43 individuals per cm^2^ of *C. harfordi* have been reported in mussel-beds [15] and up to 20 individuals per cm^2^ among serpulid tubes [33]. In California, Hewatt [15] reported that *C. harfordi* was very abundant amongst macroalgae. In the present study, only a few individuals were found inhabiting coralline turfs and those may have migrated from the adjacent oyster-bed, which had a large density of *C. harfordi*.

The abundances and distribution of *C. harfordi* found in this study in Sydney Harbour are comparable to a previous work. Bunting [18] found that *C. harfordi* was present from Balmain to Little Manly (Figure 1) using baited traps, with peak abundances in the area of Berrys Bay, which is similar to the results found here. In the present study, *C. harfordi* was, however, not found in natural oyster-beds in Little Manly. This difference might be due to the different sampling techniques used.

As discussed above, the results of the sampling done in June 2010 showed that *C. harfordi* was present in high abundances in oyster-beds. In addition, it was apparent that mussel-beds in the area were retreating and that those areas were being colonised by oyster-beds (mussel-beds were only found in three locations in June 2010, whereas two years earlier their distribution was more extensive, D. Bunting, personal communication). Therefore, the evaluation of the relationships between the densities of *C. harfordi* and the structure of the resident assemblages was done in oyster-beds. The densities of *C. harfordi* were not related with the structure of the resident assemblages in oyster-beds. These relationships may, however, be disguised by the among-location variability of assemblages. In addition, the introduction of *C. harfordi* may be influencing other ecological variables, such as body size, due to a decrease in the availability of resources. For example, the non-indigenous crayfish *Pacifastacus leniusculus* reduced total invertebrate biomass while total invertebrate density was enhanced in a Japanese marsh [34]. Future studies evaluating the impacts of *C. harfordi* should consider controlling for variability among locations by using manipulative experiments and include variables other than density to further study the relationships between *C. harfordi* and the resident assemblage.

The thickness of the oyster-bed was found to be positively related with the densities of *C. harfordi*. This is probably due to thicker oyster-beds having more habitat and shelter, as the bottom layers of oyster-beds are composed of disintegrating oyster-shells which provide with a great amount of interstitial space. Invertebrate density and richness have been found to correlate with structural complexity in a variety of habitats [35]–[38]. In contrast, organic matter was not related to the densities of *C. harfordi*. These results suggest that *C. harfordi* does not feed on deposited organic matter, or that the accumulation of organic matter in oyster-beds is very high and the consumption by *C. harfordi* is not enough to significantly reduce its amount. In addition, the lack of correlation between the amount of organic matter and the densities of *C. harfordi* might be due to its high levels of motility. The size of the sampling unit was approximately 10 cm in diameter and the densities of *C. harfordi* found in each replicate might not be representative of the amount of individuals that actually fed in that area. Future studies should evaluate the feeding habits of *C. harfordi* to further understand its ecological role in oyster-beds. In addition, the relationship between the densities of *C. harfordi* and the structure of the meiofauna (organisms between 64 and 500 μm) should be investigated since this species might be feeding on them [39].

The patterns found in this study suggest that *C. harfordi* does not affect the resident assemblage, nor the organic matter in oyster-beds. This was unexpected given the characteristics of this species (a predator and scavenger found in great abundances, as discussed above), but is consistent with similar studies, a very high proportion of which have typically failed to demonstrate significant impacts of NIS. Indeed, some of the earliest work on biological introductions by Elton [40] identified some NIS that had no apparent impact. In marine invasions, 43% of the NIS whose ecological impact was evaluated around the world showed no evidence of impacts [41]. A review of non-indigenous and cryptogenic species in Chesapeake Bay found that 29% (57 species out of 196) had little evidence of impact, while only 20% were thought to have significant ecological impacts; the remaining 51% were not investigated [12].

This study is the first published work on the ecology of the invasion of *C. harfordi*. It provides with basic knowledge on the introduction of this species, which is the first step to understand the ecology of the invasion of *C. harfordi*. Future studies evaluating the impacts of this species are necessary to design efficient management strategies and advance in the understanding of the ecology of biological invasions.
